# Improved commissioning of lung stereotactic body radiotherapy using a customized respiratory motion Phantom: a single- institutional study

**DOI:** 10.1007/s13246-025-01550-0

**Published:** 2025-05-13

**Authors:** Ashlesha Gill, Mohamed Nawar, Pejman Rowshanfarzad, Andrew Hirst, Malgorzata Skorska, Tom Milan, Nicholas Bucknell, Mahsheed Sabet

**Affiliations:** 1 School of Physics, Mathematics and Computing, The University of Western Australia, 35 Stirling Highway Crawley, Crawley, WA 6009 Australia; 2 Department of Radiation Oncology, Sir Charles Gairdner Hospital, Nedlands, WA Australia; 3 Centre for Advanced Technologies in Cancer Research (CATCR), Perth, WA Australia

**Keywords:** SBRT, Lung, Commissioning, Tumour motion, Dose blurring, 3D printing

## Abstract

Stereotactic body radiation therapy (SBRT) involves delivering high doses of radiation with geometric precision in a few hypofractionated schedules. In lung SBRT, respiratory motion is an additional concern as it could cause the delivered dose distribution to deviate from the treatment plan. Therefore, it is crucial to conduct accurate commissioning tests on a dynamic phantom. In this study, the QUASAR™ Respiratory Motion Phantom was customized using 3D-printed parts to minimize motion-induced errors in measurements. The customisations included a specialized ion chamber insert designed to move with the tumour and measure the average dose at its centre. A film insert was also developed for secure fixation, enabling precise dose verification on a static plane while minimizing the risk of friction-related damage. The quality assurance (QA) tests were performed on the plans created for phantom studies indicated that ion chamber measurements were within 1.9% of the planned dose, and film gamma analysis demonstrated pass rates over 95% using the 3%/1 mm criteria. A set of SBRT volumetric modulated arc therapy (VMAT) plans were created for a suite of test patients using both flattened and flattening filter free (FFF) 6 MV beams and utilising robust optimization. A standardized patient-specific QA protocol was used to evaluate the treatment plans of 20 test patients, yielding film gamma pass rates above 98.8%. The suggested approach, using the 3D-printed inserts, effectively mitigated dose-blurring, providing a robust tool for lung SBRT commissioning and ensuring the reliability of lung cancer treatment with SBRT.

## Introduction

Non-small cell lung cancer (NSCLC) consists of 85% of all lung cancer diagnoses [1]. Radiotherapy is the standard of care for patients with early-stage disease who are either inoperable due to comorbidities or who decline surgery [2, 3]. Over the years, adoption of refined radiation delivery techniques has led to an increased level of accuracy. Stereotactic body radiation therapy (SBRT) involves delivery of a high dose typically delivered in 1–5 fractions, resulting in a higher biologically effective dose (BED) [4]. Compared to the conventional multi-fraction treatments, SBRT minimises the local treatment failure from 31 to 14% and improves the 2-year overall survival (OS) rate from 56 to 76% for early-stage NSCLC [4]. Risks associated with the occurrence of dyspnea, esophagitis, and radiation pneumonitis are also lowered [5].

Treatment of lesions in the lung is complex due to the motion of the tumour and surrounding tissues. Several methods have been used for motion management, including 4DCT (4-dimensional computed tomography) based technique with free breathing, breath hold techniques (inhale, exhale, mid-ventilation), and free breathing gated treatment. Commissioning of SBRT necessitates particular technical pre-requisites depending on the technique used. A survey conducted by MEDPHYS Digest (through the Global Medical Physics List) in 2021 showed that 63% of the centres which responded were using the free breathing 4DCT technique for lung SBRT treatments. Consensus guidelines provide several key considerations for the choice of SBRT treatment technique [6–8]. These include the use of a linear accelerator (linac) with volumetric in-room image guidance and respiration-correlated 4DCT. Treatment planning should employ at least a type B algorithm, with internal target volume (ITV)-based contouring on the 4DCT and dose constraints and fractionation adapted to the tumour location. Rigorous quality assurance is also emphasized, with a mechanical accuracy of 1.25 mm and a dosimetric accuracy of 3% in a lung phantom [8]. End-to-end testing in a lung phantom using appropriate radiation detectors for small fields is recommended to ensure accuracy. For best practice, volumetric modulated arc therapy (VMAT) is recommended with flattening filter free (FFF) 6 MV beam, ideal for conformality as it reduces the beam-on time and the consequent intra-fraction motion [6, 9].

Lung cancer patients often suffer from comorbidities such as chronic obstructive pulmonary disorder (COPD), interstitial lung disease (ILD), bronchiectasis, and asthma, which tend to compromise the lung function. This makes them susceptible to severe radiation-induced lung toxicity (RILD) if the delivered dose exceeds the constraints specified for safe treatment [10]. The undetermined changes in respiratory motion during radiotherapy pose challenges in effectively sparing maximum volumes of healthy lung tissue and organs- at- risk (OAR). Therefore, performing sophisticated end-to-end testing is of vital importance during lung SBRT commissioning. A moving 4D phantom is recommended for this purpose [6].

The most common motion management approach for SBRT lung tumours is the free breathing ITV-based (motion-compassing) technique since the patient can breathe normally. To verify the concept dosimetrically, a phantom with tumour surrogates emulating breathing motion is ideal, but this presents difficulties in validation measurements. In commercially available respiratory motion phantoms, the film moves with the tumour which leads to a dose distribution which does not correlate with the treatment planning system (TPS) predictions. To account for this, mathematical models based on the accumulation of dose at different motion phases could be utilised, but these are not easy to develop and are not currently integrated into the routine workflows at most centres [11]. The interplay effect is another source of uncertainty that occurs when moving targets are treated with techniques involving beam modulation due to the simultaneously varying multileaf collimator (MLC) motion and tumour motion, which could lead to tumour underdosage [11]. In a conventional multi-fraction VMAT treatment, this averages out to an insignificant level due to the longer time of delivery. For SBRT-VMAT treatment with free breathing techniques, the interplay effects tend to be more significant for single fraction treatments (compared to 3–5 fractions) [12], and FFF beams due to reduced likelihood of the interplay effects averaging out over the shorter duration [13].

As shown in a previous study [14], 3D-printing solutions can be effective in managing the dose-blurring associated with a moving target during lung SBRT quality assurance (QA). A tumour insert was developed for film placement in the CIRS Dynamic Thorax Phantom. The outer shell of the insert along with the tumour was 3D-printed as a single structure, the lung volumes were manually filled with cork granules, and the insert was closed. To fit the film in the insert, the cavity needed to be wider than the film by a few micrometres, making the design prone to inadvertent air gaps. In the current work, the inserts were designed with an improved configuration, which could also be fabricated without the need to add non-printable components. In addition, the principle was extended for point dose measurements.

The present study details the commissioning of lung SBRT for the free breathing ITV-based technique as carried out at a single cancer centre, with focus on strategies to improve the initial phantom-based testing. In order to address the dosimetric verification issues, two novel custom 3D-printed tumour inserts were designed for a commercially available respiratory motion phantom (QUASAR™ Respiratory Motion Phantom) consisting of two separate holders, one tailored to film dosimetry and another to a small volume ionisation chamber. There are difficulties with the commercially available dosimeter inserts as the film moves with the tumour, and the insert for small ion chamber does not include a tumour. The proposed 3D-printed designs provide an efficient mechanism to secure the film and eliminate its movement while avoiding undesired air gaps, and the insert for small size ion chamber contains a tumour which moves with it. The proposed approach also offers flexibility for custom designs to accommodate various dosimeter models, solid tumour diameters, and ease of developing new custom made inserts at low cost. In this study, after the concept of the technique was dosimetrically verified, practice SBRT plans were created, delivered, and analysed using the routine departmental protocol for patient specific QA. The technique was released for clinical use after preparing the required protocols for imaging, treatment planning, QA, and delivery.

## Methods

Computed tomography (CT) images were acquired using an Aquilion LB CT scanner (Canon Medical Systems, Otawara, Japan). The Varian Real-time Position Management (RPM) system was used for 4DCT acquisition (Varian Medical Systems, Palo Alto, USA).

Clinical guidelines for SBRT treatment planning and delivery workflow were reviewed by examining international guidelines and methods implemented across other Australian centres [4]. As a result, it was decided to start with the free breathing ITV-based technique, which consisted of treatment planning on the average of ten phases of 4DCT images. The flattening filter-free 6 MV (6-FFF) beams were preferred due to faster delivery (1400 MU/min delivery) and less possibility of intrafractional motion; however, planning was also performed using flattened 6 MV beams for tumours with a larger range of motion (> 1 cm). Flattened beams, delivered at 600 MU/min, were chosen for tumours with larger range of motion to enable treatment delivery over a longer duration, thus minimizing the potential impact of motion-related uncertainties, mainly resulted by the interplay effect. The ITV was ideally created by summing the GTV volumes on each phase of the 4DCT or, in suitable tumours away from anatomical barriers, contouring on the maximum intensity projection image and checking this against each phase. A 5 mm expansion of ITV was used as the planning target volume (PTV).

Treatment plans were created using Eclipse treatment planning system (Varian Medical Systems, Palo Alto, USA) with AcurosXB dose calculation algorithm (v 15.6). The dose values predicted by treatment planning system were used as reference for comparison with measurements.

A QUASAR™ Respiratory Motion Phantom (Modus Medical Devices, ON, Canada) was used for dosimetric verification of the method. Customized inserts for the phantom were designed and 3D-printed for dosimetric verification of the technique.

All measurements were carried out on a TrueBeam linac (Varian Medical Systems, Palo Alto, USA) using pieces of Gafchromic™ EBT3 film (Ashland Inc., Covington, KY, USA) and an EPSON 12000XL scanner (Nagano, Japan). Point dose measurements were performed with a CC04 ionization chamber (IBA Dosimetry, Schwarzenbruck, Germany) in conjunction with a PTW UNIDOS Webline (Freiburg, Germany) electrometer, which were cross calibrated against the department’s reference dosimeters (regularly calibrated at ARPANSA).

It must be noted that the accuracy of the algorithm and the testing of simple open fields were already verified as part of the commissioning of conventionally fractionated VMAT for lung lesions, which were performed on a more basic design of these inserts; therefore, the results have not been included in this work.

### 3D-printed detector inserts for the QUASAR Phantom

**Fig. 1:**
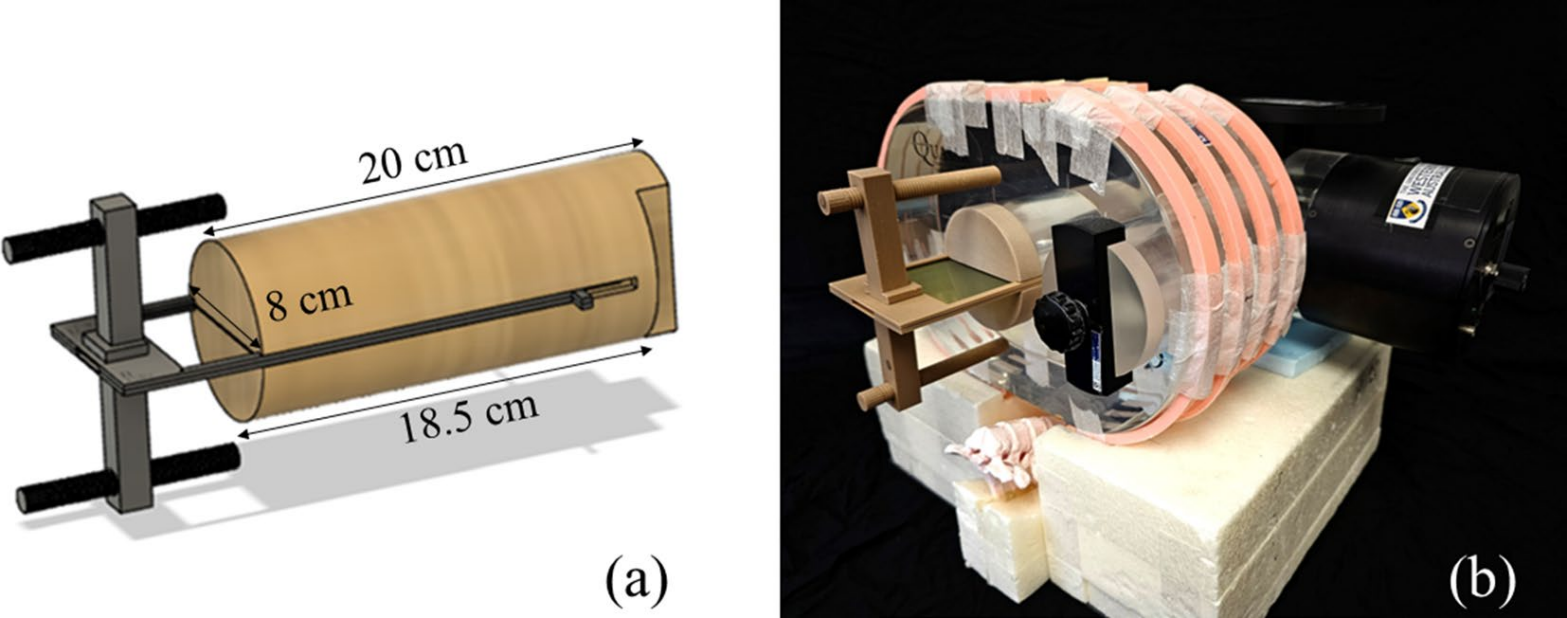
(**a**) CAD model designed in Fusion 360 for the film holder (**b**) 3D-printed product fitted inside the QUASAR phantom (The ribs and spine were added as part of a previous project)

In this study, the QUASAR phantom was used for dosimetric validation of the planning technique. This commercially available phantom consists of an oval Perspex body, a motion assembly that can facilitate simulation of respiratory waveforms and an opening to place the inserts provided with the phantom for dosimeter placement. To address the limitations of QUASAR phantom for dosimetric evaluation using film (proof of concept), custom-made 3D-printed inserts were designed in a computer-aided design (CAD) software (Fusion 360, Autodesk, San Rafael, CA, USA) to enable the tumour to move with the body of the insert, while keeping the film static between the two halves of the insert (Fig. 1).

The insert was designed as a cylinder featuring a thin slot along its length for film insertion. Inside the lung insert, a 3 cm diameter spherical tumour was split into two halves, with each half located on opposite sides of the film. The holder was designed to accommodate 18.2 cm × 7.5 cm pieces of Gafchromic™ EBT3 film. To protect the dosimetry film from potential friction-induced damage during motion, the film slot was thick enough to contain two additional cushioning layers within the film insert, one positioned anteriorly and the other posteriorly. A fixation device was designed at the inferior edge of the film holder to keep it firmly fitted in the body of the phantom (Fig. 1). The insert was 3D-printed using a Prusa i3 MK3S printer (Prusa Research, Czech Republic) incorporating polylactic acid (PLA) filament (X3D Filament, Western Australia), specifically 93% infill wood-PLA to achieve lung-equivalent Hounsfield units (HU) in the body of cylinder and 24% infill wood-PLA to attain soft tissue-equivalent HU numbers for the tumour. A nozzle temperature of 200 °C, a bed temperature of 60 °C, a flow rate of 100%, a print speed of 50 mm/s and a layer height of 0.2 mm were selected based on general PLA recommendations. These parameters were further fine-tuned to ensure mechanical accuracy, and a larger nozzle size was used to aid printing with wood-PLA.

**Fig. 2:**
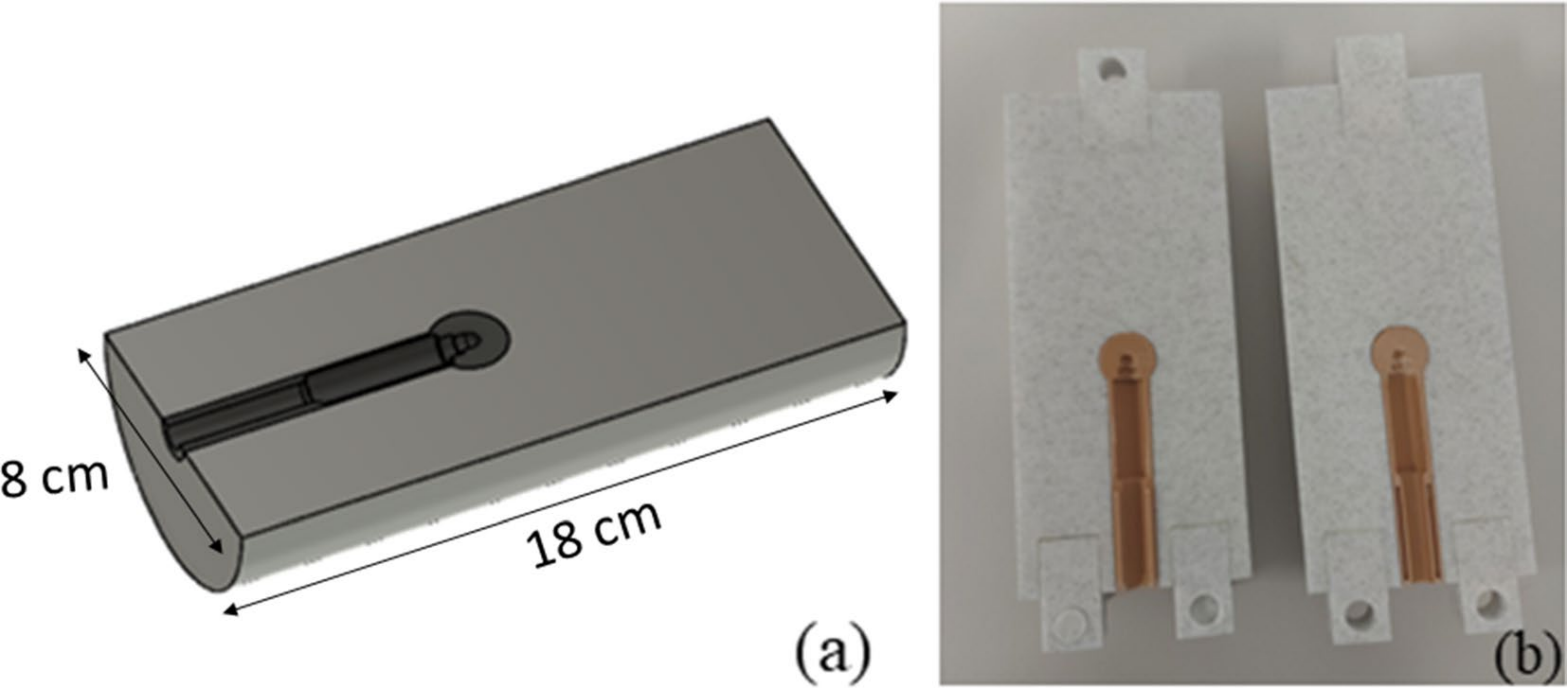
(**a**) CAD model for the ion chamber insert designed in Fusion 360 (**b**) 3D-printed insert

The ion chamber insert was designed for point dose measurements inside a tumour. The cylinder was 18 cm long with 8 cm diameter. A cavity was modelled with the size and curvatures suitable to fit a CC04 chamber in the centre of a spherical 2 cm diameter tumour (Fig. 2). This way, the ion chamber moved with the tumour and measured the dose with greater precision than the commercial insert, given that the chamber was specifically created to house the CC04 chamber in the middle of a small tumour such that the chamber moved with the tumour.

The insert was designed and constructed in two halves such that it could be screwed together as facilitated through the 3D-printed structure and fit in the phantom. In this design, the detector moved with the insert; therefore, the measured point dose represented the actual dose received at the centre of the tumour. The 3D printing was performed using similar print settings as the film insert, with the only difference being the use of 28% infill marble-PLA for the lung-equivalent region of the insert. Both inserts were CT scanned to ensure structural integrity and to confirm tissue equivalency by analysing the HU values.

Two sets of 4DCTs were acquired of the QUASAR phantom each with one of the above inserts and the detectors in place. SBRT plans were created on the average of the 10 phases, delivered and compared with the TPS calculated dose distributions: 2D comparisons on the films and point dose comparisons with the CC04 chamber.

### Treatment planning and QA on test patients

**Fig. 3:**
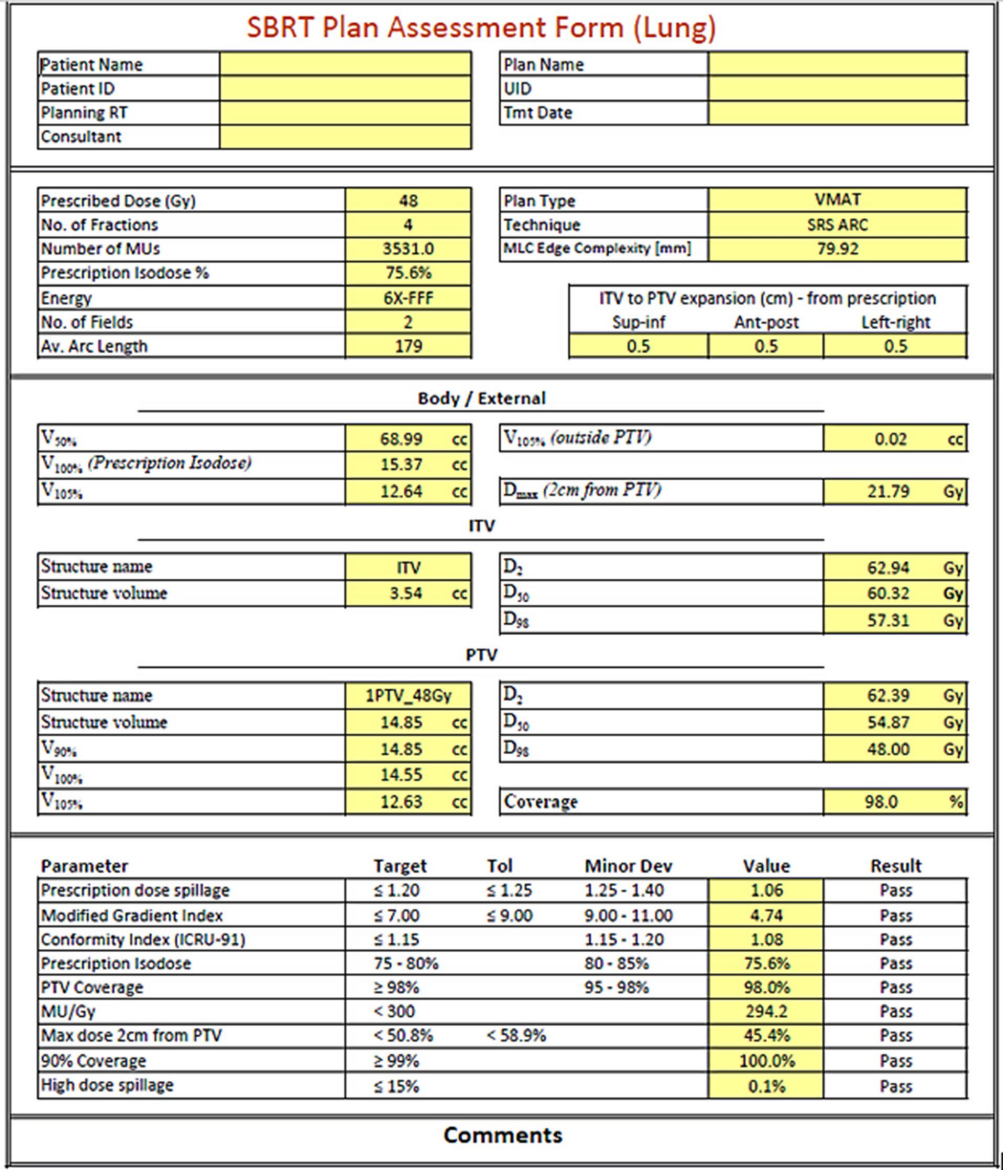
A sample anonymized plan quality check sheet automatically created for one of the test patients

Twenty existing lung patients with peripheral tumours [15] with maximum diameter of 5 cm who had been treated with conventionally fractionated VMAT employing the ITV-based free breathing technique were selected, anonymised, and used for an SBRT planning study by radiation therapists experienced in SBRT planning. The dose fractionation used was either 48 Gy in 4 fractions or 54 Gy in 3 fractions depending on patient-specific considerations. Plan qualities were checked by physicists, and as a result, the optimal settings in the TPS were decided. Robust optimization techniques were applied to keep the leaves as open as possible minimizing the interplay effect. The robust techniques included the use of MU objective, setting a complexity measure up to 300 MU/Gy, use of one or two coplanar 200 degree arcs and ensuring the aperture shape controller (ASC) was set on very high. A high ASC reduces the local curvature of the aperture, determined by the positions of adjacent MLC leaf tips shaping the same spatially continuous target projection. MU objectives can be set so that MU values are controlled and cannot go above a certain level, thus limiting MLC leaf modulation. Setting these parameters even made a visible difference to the MLC opening in beam’s eye view as they were keeping the leaves open and the field less modulated. Dose constraints were checked on each plan against a number of reference documents, including eviQ [15], SAFRON II clinical trial [16], UK Consortium 2022 [17], and AAPM Task Group 100 [18]. A script was written to calculate and record a summary of the plan quality parameters on a sheet (Fig. 3). This sheet is created for each patient and uploaded on the patient’s chart in the record and verify system.

## Results

### Phantom study: film measurements

Film measurements in phantom were compared with TPS predictions using gamma analysis in an in-house developed software. Results were analysed in absolute global mode using a (3%, 1 mm) criteria for both 6FFF and flattened 6 MV beams. Details are given in Table 1. Due to the limited lateral space for film insertion inside the phantom, measured dose profiles suddenly drop in the figures and the gamma thresholds were limited to these levels.

**Table 1:** Gamma pass rates for the two SBRT plans tested in Phantom (6FFF and flattened 6MV) using (3%, 1 mm) and (3%, 2 mm) criteria

Beam Energy	(3%, 1 mm)		(3%, 2 mm)	
	Field 1	Field 2	Field 1	Field 2
6 FFF	99.5%	97.8%	100%	100%
Flattened 6 MV	95.2%	99.5%	98.2%	100%

All fields showed gamma pass rates above 95% with (3%, 1 mm) and (3%, 2 mm) criteria.

### Phantom study: ion chamber measurements

Point dose measurements made with the CC04 chamber were compared to the expected value from the planning system in Table 2. Two plans were tested, one for each energy. Each plan was delivered 5 times.

The mean relative difference ± 1 standard deviation values were 1.2%±0.4% and 0.7%±0.2% for the plans with 6FFF and flattened 6MV beams, respectively.

**Table 2:** Comparison of the CC04-measured point doses in the moving tumour with the expected TPS-calculated values

Measurement	6-FFF MV			6 MV		
	TPS Dose (Gy)	Measured Dose (Gy)	Relative Difference	TPS Dose (Gy)	Measured Dose (Gy)	Relative Difference
1	14.504 ± 0.102	14.345	1.1%	14.546 ± 0.055	14.479	0.5%
2		14.233	1.9%		14.433	0.8%
3		14.355	1.0%		14.423	0.8%
4		14.345	1.1%		14.394	1.0%
5		14.369	0.9%		14.467	0.5%

### Plan quality checks and patient-specific QA

The SBRT test plans, created on patient CT datasets, successfully met the plan quality criteria following their evaluation against international guidelines [15–18]. Physicists reviewed the plans and provided feedback or requested replan where needed, then performed patient-specific quality assurance (QA) on these test plans in line with the routine departmental protocol, utilizing stationary film and point dose measurements with a CC04 ionization chamber. The average gamma pass rate on films was 98.8 (6-FFF MV) and 98.9 (flattened 6 MV) using the (3%, 1 mm) criteria in absolute mode, and CC04 point dose measurements were within 2% of expected value. This confirmed the deliverability of test plans and acceptable delivered dose distributions.

Protocols were prepared for CT, planning, physics QA, and treatment imaging. Independent audit was performed using film and ion chamber measurements in the stationary CIRS thorax phantom. Point doses were within 1.5% and gamma evaluation of film comparisons showed pass rates of 98.9% for 6-FFF and 96.2% for flattened 6MV plans using the (3%, 2 mm) criteria.

## Discussion

The paper details the commissioning of SBRT for lung cancer treatment in a radiotherapy centre. Bespoke 3D-printed inserts were constructed to address the limitations of commercially available QUASAR lung phantom for dosimetric evaluation of the concept. Efficacy of these innovative changes was demonstrated through validation of the delivered planned SBRT plans created with 6 FFF and flattened 6 MV beams, the latter to be clinically used for tumours prone to larger displacement, where delivering SBRT plans over an extended duration is preferable. Optimal results were observed for both the beam types using a strict gamma criteria (3%, 1 mm) despite the uncertainties and difficulties of our film dosimetry system. The results from independent audit confirmed the ability of the department to safely deliver lung SBRT.

A previous 3D-printed tool was developed by Retif et al. [14] where an insert was modelled for film measurements in CIRS Dynamic Thorax Phantom. Although advantageous for primary immobilisation, the design lacked sufficient features to safeguard the film from damage during insertion and avoid unexpected air gaps produced around the film while the phantom moved. The gamma pass rate with the (3%,1 mm) criteria was provided per plan (not per field) and was lower than those measured in this study. Limitations of the former study were resolved in the present work by considering the film holder as a separate component of the insert, designed to open on both sides for placement of the film, which can then be securely fastened and attached to the insert. The updated design allowed a larger size of film to be used in the present study. Moreover, the previous study involved a multi-step approach incorporating both 3D-printing and non-printable lung-equivalent materials (cork granules). To enhance the cost-effectiveness and time efficiency, the present work used a direct manufacturing procedure based on filament infills that were calibrated to tissue-equivalent and lung-equivalent HU, eliminating the need to manually add the cork granules. The final insert prints resulted in -705 ± 10.7 HU for lung tissue and 5.5 ± 10.8 HU for the tumour, meeting the desired tissue- equivalency as checked on the real patient CT data. The ~ 11 HU variation was considered acceptable uniformity as it would not result in a notable dose difference in Eclipse calculations.

Film measurements provide a comparison of 2D dose distribution in a plane but do not represent the dose received at a point in the tumour. Therefore, the additional insert for the CC04 chamber was necessary to provide information about the actual dose to the moving target. Considering the small size of the CC04 thimble and difficulty of contouring it, the point dose differences (averaged over the thimble volume) were not more than 1.9% in any of the measurements which was reassuring as it falls below the maximum allowable threshold of 3% [8].

The study addresses the literature gap existing for end-to-end tests of SBRT-VMAT lung treatment plans for a moving target using a dynamic anthropomorphic phantom. Previous studies have reported point dose deviations between measured and Eclipse-calculated doses using the AcurosXB algorithm. These deviations were as high as 5% for a 2 cm target and 10% for a 1 cm target with 6-FFF MV [19]. For a 2 cm target with 6 MV, deviations were up to 2.5% [20]. For a 0.75 cm central lung target, deviations were up to 3.2%, based on a median of data from 12 institutions. Most of these institutions used 6 MV and type A algorithms, with only one institution using 6-FFF MV and the AcurosXB algorithm [21]. The results from the present study are consistent with the above and expand the work to QUASAR lung phantom, which wasn’t used in the previous studies. However, since this was just performed as proof of concept, only a 2 cm target was used for point dose measurements which could provide sufficient amount of scatter while not being overly large. The custom ion chamber insert serves as a cost-effective alternative to commercial options, which are typically not designed to accommodate the tumour around the ion chamber. Historically, dose-blurring due to target motion has been corrected using mathematical convolution algorithms that represent the final dose for a moving target to be equivalent to the convolution of dose distributions intended for a hypothetically static target, employing a Gaussian motion kernel [22]. This method is limited to phantoms with simple geometries and linear motions [23, 24]. Alternatives based on estimating target motion after dividing into position bins and modelling isocenter shifts for the assumed sub-beams [25], and averaging the summation of dose calculated with the specific isocenters at different motion phases [11] have been proposed. These solutions may not be available at all centres and there is also room for improvement due to the limited complexity for reconstructing a realistic dose from using simplistic ITV-based planning. The suggested 3D-printing approach of using a phantom setup resistant to dose-blurring effect, eliminates the need to correct it in the treatment planning system.

The proposed phantom insert maintains the film in a static state, which allows for dose comparison with the TPS based on a single 4DCT for lung SBRT. However, to measure the actual dose delivered to the tumour, a moving film would be more appropriate. In the ion chamber insert, the dosimeter moves with the tumour, providing a useful method for cross-validating the dose measured by the film. Due to the density differences between the lung and the tumour, the tumour inherently absorbs a higher dose even when in motion. This results in a smaller margin of error and renders dose discrepancies negligible.

This study was only performed as proof of concept and the number of tested phantom plans was quite limited. Ideally, testing a larger number of tumour shapes and sizes moving in various motion patterns would be much more desirable. However, it requires 3D printing a number of inserts and allocation of resources for time-consuming labour-intensive measurements, which were not possible in the present study, considering the high workload, limited time for the project, and limited resources in a busy radiotherapy department.

Patient-specific test plans could also be recalculated and tested on the phantom with custom made inserts for each case, but again it was not feasible in this study considering the above-mentioned limitations. Using the phantom is quite cumbersome and although it may sound desirable for patient QA measurement, it may not be smooth for routine use, but more suited for commissioning measurements.

The commercial phantoms used for lung SBRT commissioning/QA move sinusoidally in the superior-inferior direction. Although patient’s breathing motion is most noticeable in this direction, the realistic movement may be complex. Development of sophisticated deformable thorax phantoms with six degrees-of-freedom can aid in better representation. The evaluation of 3D dose distribution was not needed in this study, but future work can include extending the 3D-printing strategies for accurate gel dosimetry or 3D dose reconstruction from multiple parallel films for the moving target. Moreover, a clinical protocol can be developed to reproduce the proposed 3D-printing methodology for different models of films and ionisation chamber specific to the phantoms in various departments.

## Conclusion

This study presents the methodology and design principles for 3D-printing of bespoke tumour inserts for commercial phantoms to cater to the requirements of radiation dosimeters with particular shapes and sizes. The method can efficiently mitigate the dose-blurring effect during the feasibility testing of SBRT technique using film dosimetry in a moving phantom. It is much simpler than accounting for it using complicated mathematical models. This was simply achieved by maintaining the film stationary relative to the moving tumour while safeguarding it from potential damage during the dynamic phantom motion. The ion chamber insert helped by ensuring the accuracy of point dose delivery in the moving target. Test plans on patient CT sets met the criteria for patient specific QA and the technique was successfully commissioned in the department.
